# Age-Related Differences in the Reliance on Executive Control in Working Memory: Role of Task Demand

**DOI:** 10.1371/journal.pone.0145361

**Published:** 2015-12-23

**Authors:** Michel Isingrini, Lucie Angel, Séverine Fay, Laurence Taconnat, Patrick Lemaire, Badiâa Bouazzaoui

**Affiliations:** 1 Centre de Recherches sur la Cognition et l’Apprentissage, François Rabelais University of Tours, Tours, France; 2 Centre National de la Recherche Scientifique, Poitiers & Tours, France; 3 Laboratoire de Psychologie Cognitive, Aix-Marseille University, Marseille, France; 4 Institut Universitaire de France, Paris, France; University Medical Center Goettingen, GERMANY

## Abstract

We examined the hypothesis that age-related differences in the reliance on executive control may be better explained by variations of task demand than by a mechanism specifically linked to aging. To this end, we compared the relationship between the performance of young and older adults on two executive functioning tests and an updating working-memory task with different load levels. The results revealed a significant interaction between age, task demand, and individual executive capacities, indicating that executive resources were only involved at lower loads in older adults, and only at higher loads in young adults. Overall, the results are not consistent with the proposition that cognition places greater demand on executive control in older adults. However, they support the view that how much young and older adults rely on executive control to accomplish cognitive tasks depends on task demand. Finally, interestingly these results are consistent with the CRUNCH model accounting for age-related differences in brain activations.

## Introduction

Although it is well established that executive functioning declines during aging, an issue of current interest is that older adults appear to rely more on executive control than young adults when faced with a demanding task [1–3]. Recent studies in the domains of memory and reasoning have demonstrated that executive function measures correlate more with the performance of older adults than with that of young adults [4–8]. Accordingly, some authors have postulated that cognitive tasks place greater demand on executive control resources in older than in young adults, and that this phenomenon may correspond to an aging-specific mechanism to compensate for age-related differences (a hypothesis referred to as a “*shift from automatic to controlled forms of processing with advancing age”*) [4, 9, 10].

Greater correlations between executive functioning measures and the cognitive performance of older adults are consistent with recent functional neuroimaging data on age-related differences in brain activations. Larger brain activations, referred to as *over*-activations, have been found in older than in young adults, predominantly in brain areas known to underlie executive functioning, such as the prefrontal cortex (PFC) and the parietal cortex (PC). Age-related brain over-activations have been observed in many cognitive tasks, such as episodic memory, perceptual, and reasoning tasks, as well as tasks involving working memory (WM), the domain of the present study [1, 11, 12]. This phenomenon has generally been interpreted as a specific neural response associated with aging to overcome the impact of smaller neural capacity and/or efficiency ([1, 13–15] for a review). By comparing these neuroimaging data with behavioral data regarding the greater reliance on executive functioning in older adults, some authors have inferred that this additional neural recruitment in cortical areas, classically associated with executive functioning, may reflect a greater involvement of executive control processes in order to limit age-related cognitive decline [4, 5, 8, 10, 11, 16].

However, an important outstanding question concerns whether this greater involvement of executive control is specific to older adults or can also occur in young adults when the task demand exceeds a certain threshold. Given the reduced resources of older adults, their reliance on executive control would start to take effect at a lower level of task demand than that of young adults. To test this hypothesis, the three variables—age, executive control, and task demand–must be varied within the same experimental design. The notion that task demand is a crucial factor in the involvement of executive resources is consistent with the CRUNCH model (*Compensation-Related Utilization of Neural Circuits Hypothesis*), which assumes that older adults’ greater reliance on prefrontal cortical resources is due to task demand, rather than to aging *per se* [17]. This model suggests that greater task demand increases recruitment in some cortical regions among both older and younger adults, but at different levels of load. Strong evidence for this prediction has recently been found in functional neuroimaging studies showing that age-related differences in the brain activation of the prefrontal cortex associated with working memory follows a particular profile, with over-activation in older adults at lower load levels, disappearing at higher levels, and appearing only at higher load levels in young adults [18–23]. Given the strong involvement of the prefrontal cortex in executive functioning [1], an interesting question is whether a similar pattern could be observed in the reliance on executive control.

The main purpose of this study was to test the hypothesis that increased reliance on executive control corresponds more to a general mechanism occurring when both young and older adults are faced with heavy task demand relative to their respective resources. We assume first that age-related differences in the reliance on executive functioning depend mainly on task demand, and secondly, probably due to their reduced capacity, that older adults will be more likely to show greater reliance on executive functioning at lower memory loads, whereas young adults will show this reliance at higher loads. We examined this issue by measuring the performance of young and older adults on an Updating Working-Memory Task (UWMT) with six different load levels, and by assessing executive functioning using two tasks, namely the Wisconsin Card Sorting Test (WCST) and the Initial Verbal-fluency Test (ILFT).

We expected to observe a significant three-way interaction between age, level of task demand, and executive functioning, providing statistical confirmation of differences between young and older adults regarding the level of executive functioning involved at each level of task demand. Accordingly, a relationship between Updating Working-Memory Task and executive functioning performance should begin to be significant at lower levels of demand and continue to be significant at higher levels in older adults, and should be significant only at higher levels of demand in young adults. However, regarding older adults, a possible alternative result is that the working memory and executive functioning relationship would become non-significant at higher load levels. This would occur if, across memory-load conditions, this relationship follows the pattern predicted by the CRUNCH model for prefrontal cortex recruitment, whereby older adults would have reached their limit in the involvement of executive resources at higher load levels.

## Material and Method

### Participants

The sample comprised 101 participants divided into two age groups: 48 young adults aged between 25 and 45 years (M = 32.52, SD = 6.63) and 53 older adults aged between 60 and 80 years (M = 70.83, SD = 5.79). The number of males and females was approximately the same in the two age groups, with 19 men and 29 women in the young group, and 22 men and 31 women in the older group. The sample sizes for each group were planned for approximately fifty participants, representing the data-collection stopping rule. This was based on indications of a previous study [24] comparing young and older adults on an updating working-memory task, and also to ensure sufficient power to demonstrate a significant effect of age on the updating working-memory task and correlations between this task and executive measures within each group. We tested 48 young participants and 55 older participants. Two older participants were excluded from the study for their difficulty performing the UWMT. The study was approved by the Institutional Review Board of the Cognition and Learning Research Center (CeRCA-UMR CNRS 7295) of the University of Tours and the University of Poitiers. Given that no invasive technology was used, and that only adults were involved, and because the data were analyzed strictly anonymously, the authors considered that participants could give their informed consent orally. Consent was recorded in a research logbook alongside each participant’s number. This procedure was approved by the authors’ Institutional Review Board.

Participants performed the Mill-Hill Vocabulary test [25]. A significant effect of age was found in educational level, younger adults having completed more years of education (M = 14.21, SD = 3.21 for the young group; M = 12.00, SD = 2.93 for the older group; [t(99) = 3.62, p < .001]). No significant difference was found in vocabulary skills, suggesting that, despite the difference in the number of years of education, the two groups had a similar level of general knowledge (M = 22.73, SD = 4.49 for the young group; M = 23.41, SD = 4.50 for the older group); [t(99) = -0.81, p = .42]). All participants were volunteers, and none were taking medication likely to affect their cognitive abilities. The older adults scored above the 27 cut-off point on the Mini-Mental State Examination [26], indicating that none suffered from dementia or cognitive impairment.

### Materials and design

Participants carried out an updating working-memory task (UWMT) and two executive function tests (WCST; ILFT).

### Working Memory task

#### Updating Working-Memory Task (UWMT) [27, 28]

The UWMT was chosen as the target working-memory task because it enables working-memory performance to be measured at different levels of updating load, easily manipulated through the number of updating operations required in each trial.

In the Updating Working-Memory Task, participants listen strings of items of unknown length, and then have to recall a specific number of items in the correct order. In the version used in this study, participants were presented with lists of *7*, *8*, *9*, *10*, *11*, or *12* consonants. They had to recall the last six consonants of each list in the order of their presentation. This involved holding in memory the first six items and thereafter updating the items stored in memory by dropping the “oldest” item and adding the most recent one. This updating process must be repeated for each additional item. This procedure requires participants to make one to n updates with subsequent serial recall, here from *1* to *6*. Participants were not told the length of each list before presentation; the experimenter indicated the end of the list. Participants were asked to rehearse silently and to remember only the last six items, which they then had to say aloud in the correct order after presentation of each list. Sequences sounding like words and abbreviations were avoided, and the lists were presented in a randomized order. The updating task score was the number of correct items recalled in the correct order, with a maximum score of *6* in each of the *6* load conditions (total maximum score = 36).

Note that the Updating Working-Memory Task was designed to tap “short-term storage” and “updating” [28]. However, “updating” is also classically postulated to be a distinct executive function involved in regulating behavior [28]. Thus, the Updating Working-Memory Task can be expected to share some variance with tasks designed to measure executive control, which could partly mask the variations we expected to observe in correlations between the UWMT and executive function tasks (i.e., WCST, ILFT). Some authors have observed that it can be difficult to clearly define and separate working memory and executive functions (e.g., see [2, 29]), since these two abilities share similar processes. However, we chose the Updating Working-Memory Task because we assumed that this possibility would be low, as the Wisconsin Card Sorting Test-Modified and the Initial Letter-Fluency Test have been defined as complex executive tasks capturing multiple executive functions, including “mental set shifting or mental flexibility”, “inhibition”, “generation”, “monitoring” and “categorization”, but not specifically “updating” [30].

### Executive function tests

The following executive function tests were chosen because they are commonly used as measures of multiple frontal-executive functions [28, 30, 31].

#### Wisconsin Card Sorting Test-Modified (WCST) [32]

The WCST is a standardized test for measuring set formation and attention shifts. Participants have to sort cards containing multidimensional drawings into different categories on the basis of minimum positive and negative feedback from the experimenter. This test is a goal-oriented task assumed to measure multiple executive or frontal functions [33]. It is often used to measure set shifting or mental flexibility, but it is also assumed to measure inhibition of previous task sets, problem solving, abstract thinking, and concept generation (see 30). A structural MRI study [34] and a study of patients with frontal lobe lesions [35] demonstrated that the Wisconsin Card Sorting Test-Modified is associated with prefrontal cortex regions. A significant effect of age on performance is classically found [36, 34, 31]. Moreover, a study [34] demonstrated that shrinkage of the prefrontal cortex mediates an age-related decrease in performance. The measure reported in the present study is the number of correctly achieved categories.

#### Initial Letter-Fluency Test (ILFT) [37]

The ILFT is a standardized test in which participants are asked to produce as many words as possible beginning with the letters F, A, and S in one minute for each letter. The score is the total number of correctly produced words. It is classically described as a complex multifaceted executive task that is reliant on aspects of executive functioning, such as generating and maintaining the search strategies needed to activate relevant responses and to suppress irrelevant ones [30]. It is therefore believed to measure the executive functions associated with inhibitory functioning, memory monitoring, and switching between retrieval strategies [30]. Performance is impaired in patients with frontal and executive dysfunctions [33]. Functional imaging studies have demonstrated that phonemic verbal fluency is associated with an increase in left dorsolateral prefrontal cortex activity [38]. A significant effect of age on performance of the ILFT has been found [36, 39], although some studies reported no such effect [40].

#### Executive functioning composite index (EFCI)

We also calculated an executive composite score for each participant to assess overall executive functioning. To do so, we averaged the standardized z-scores of the two executive tests. To justify this composite score, we carried out a factor analysis including the Wisconsin Card Sorting Test-Modified and Initial Letter-Fluency Test measures, which yielded a single factor (accounting for 78% of the explained variance) to which both measures contributed significantly. This executive composite score was of particular usefulness in the confirmatory General Linear Model (GLM) analysis, by allowing the executive measures to be summarized with a single variable.

### Results

Three analyses were carried out, using the STATISTICA software, version 12. First, using Student’s t test, we examined the effects of age on each executive measure (WSCT, ILFT, EFCI). Second, a General Linear Model (GLM) analysis was carried out to identify main effects of age group, load level, individual executive level, and interactions between these factors. By examining the three-way interaction between these three factors, we specifically tested the main statistical hypothesis of the present article, namely that level of executive functioning determines performance on the Updating Working-Memory Task, but with differences between young and older adults in relation to task demand. Third, to illustrate how the relationship between working memory and executive control varies as a function of age and task demand, we computed correlations between performance at the six load levels in the Updating Working-Memory Task and each measure of executive functioning, in both young and older adults.

### Age group-related differences in executive functioning

To examine age group-related differences in performance on each of the three executive function measures (WCST, ILFT, EFCI), we computed Student’s t tests. Mean scores are summarized in Table 1. The analyses show that age group had significant effects on the Wisconsin Card Sorting Test-Modified and Executive functioning composite index, but not on the Initial Letter-Fluency Test, although young adults outperformed older adults in this task.

**Table 1:** Means and Standard Deviations of executive functioning measures in young and older adults.

	Young (n = 48)	Old (n = 53)	t (1,99)
*Executive functioning*			
ILFT	26 (4.68)	24.47 (6.83)	1.30 (p = .20)
WCST	5.75 (0.48)	5.19 (0.76)	4.37 (p < .001)
EFCI	0.39 (0.56)	-0.09 (0.93)	3.11 (p = .002)

ILFT: Initial Letter-fluency Task

WCST: Wisconsin Card Sorting Test

EFCI: Executive Functioning Composite Index

### Influence of executive level on UWMT as a function of age group and load level *(GLM analysis)*

Consistent with the mixed design planned for this study, performance data for the Updating Working-Memory Task were analyzed in a 2 (age group: young and old) x 6 (load level: 1 to 6) x individual executive score General Linear Model analysis (GLM), with individual executive level as continuous predictor. Note that the type of GLM analysis we selected, based on the linear regression model, involves the ANOVA. It is used to analyze models with all combinations of continuous (as individual executive level) or categorical (as age-group) predictors when applied to quantitative scales, and it allows the effect of repeated measures (as load variation in this study) to be calculated.

**Fig 1:**
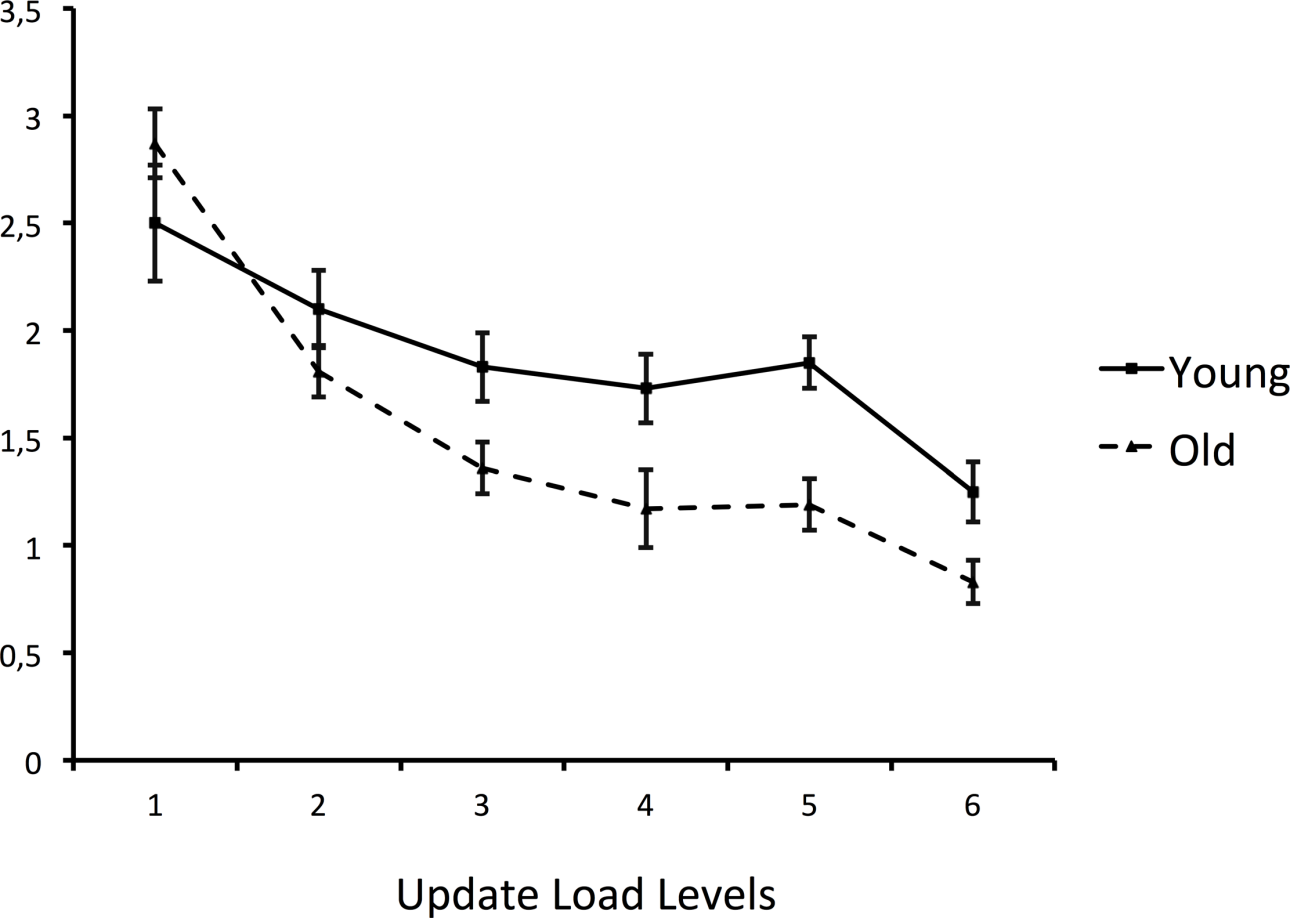
Updating Working Memory task (UWMT) performance (means and standard errors) as a function of updating load level and age group. Performance data on the Updating Working Memory Task (UWMT) were collected at each updating load level.

The analysis revealed a significant effect of age group indicating that older adults performed less accurately than young adults (F(1,97) = 6.54, MSe = 2.68, η ^2^_p_ = 0.06, p = .01), and a significant effect of load level indicating that performance decreased with increasing load (F(5,485) = 37.97, MSe = 0.84, η ^2^_p_ = 0.28, p < .001). The interaction between age group and load level was significant (F(5,485) = 4.11, MSe = 0.84, η ^2^_p_ = 0.04, p = .001), indicating that the performance of older adults decreased more than that of young adults with increasing memory load. Mean scores are summarized in Fig 1.

**Fig 2:**
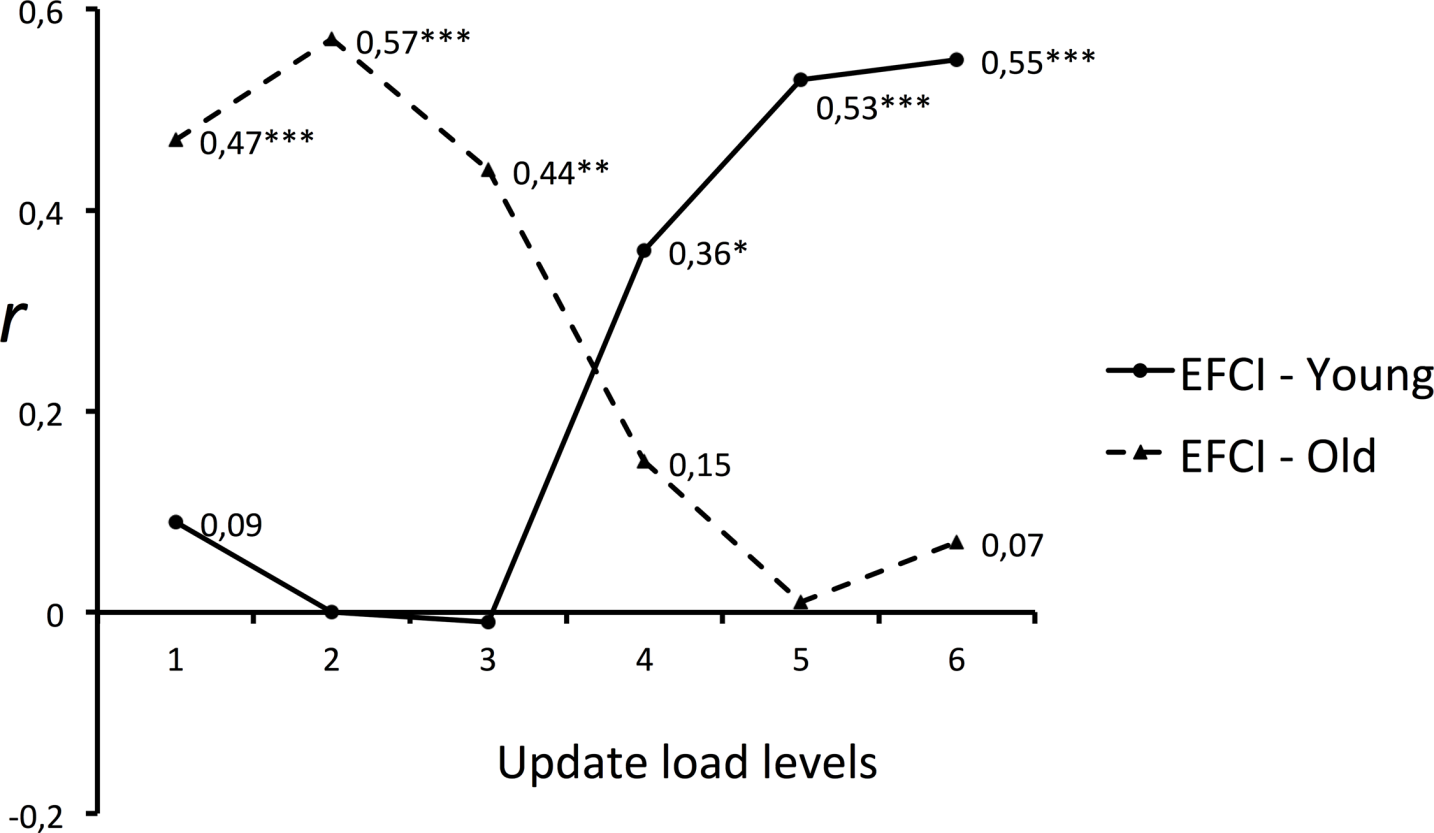
Partial Pearson correlations between Updating Working Memory task (UWMT) performance and executive functioning (EFCI) as a function of updating load level and age group. We collected performance data for the Updating Working Memory Task (UWMT) at each updating load level, and analyzed correlations with executive functioning performance, as indexed by the executive functioning composite index (EFCI). Only significant correlations with confidence intervals that did not include 0 are labeled. EFCI: Executive Functioning Composite Index. Note: * = p < .05; ** = p < .01; *** = p < .001

The analysis also revealed a significant effect of executive level (F(1,97) = 20.52, MSe = 2.68, η ^2^_p_ = 0.17, p < .001), indicating that performance decreased with lower executive levels, and no significant interaction between executive level and either age (F(1,97) = 0.38, MSe = 2.68, η ^2^_p_ = 0.01, p = .54) or load level (F(5,485) = 0.96, MSe = 0.84, η ^2^_p_ = 0.01, p = .44). Interestingly for the present goal, the analysis yielded a significant three-way interaction between age, load level, and executive functioning (F(5,485) = 5.24, MSe = 0.84, η ^2^_p_ = 0.05, p < .001). As illustrated by the correlations analysis below (Fig 2), this interaction is due to the fact that the pattern of reliance on executive functioning clearly differs between the two age groups, depending on the load level.

### Correlations in young and older adults

To illustrate and explain the significant three-way interaction between age, load level, and executive functioning yielded by the GLM analysis, we performed partial Pearson correlations in young and older groups separately, with age introduced as a continuous covariate. This was done on relationships between performance at the six updating levels of the Updating Working-Memory Task and the composite executive functioning measures (EFCI) in each age group. Fig 2 summarizes these correlations. The general pattern of correlations illustrates the result of the ANOVA. It shows that the three-way interaction is due to the fact that correlation patterns of the Updating Working-Memory Task with the executive functioning between young and older adults are different. In the young group, the Updating Working-Memory Task performance was positively correlated with the measure of executive functioning only at the highest load levels *(4*, *5*, *and 6)*. In the older group, these correlations were significant only at the lowest load levels *(1*, *2*, *and 3)*. These results show that the Executive Functioning Composite Index measure (EFCI) and the working memory task measure are related and vary in the same way; however, this was true only at the lowest memory load levels for older adults and only at the higher memory load levels for young adults. The calculation of the 95% confidence intervals confirmed the pattern of p values. For all significant r correlations, the confidence intervals did not include 0, whereas this was the case for all non-significant r correlations.

## Discussion

The aim of the present study was to examine the hypothesis that age-related differences in the reliance on executive control might be better explained by variations in task demand than by a mechanism specifically linked to aging [1–10]. To this end, we compared the relationship between performance on two executive function tests, as indicators of executive control resources, and performance on an updating working-memory task with different load levels, in young and older adults. We also assumed that the specific profiles of reliance on executive control of young and older adults as a function of task difficulty would be close to the CRUNCH pattern postulated to explain reliance on the prefrontal cortex [17].

Before looking at the results directly referring to these assumptions, we can note that our data reveal the classic aging pattern, with young adults outperforming older adults on the Updating Working-Memory Task, the Wisconsin Card Sorting Test-Modified, and on the Executive Functioning Composite Index. In this study, no age effect was observed on the verbal fluency task (ILFT), which is in line with previous studies [40]. Overall, our results also confirm a significant relationship between the updating process in working memory and executive functioning, which can be explained by the inhibition and switching capacities involved in the Wisconsin Card Sorting Test-Modified and the Initial Letter-fluency Task. Achieving update operations involves the capacity to inhibit to-be-forgotten items, and to switch attention to new items. Finally, the classic robust Age x Complexity interaction found across the board of cognition was replicated, as the performance of the older adults on the updating working memory task declined with increasing load more than that of young adults.

Regarding the main hypothesis tested in this study, the pattern of the relationship observed between working memory and executive functioning when load level was varied, in young and older adults, is consistent with the hypothesis that greater reliance on executive control mainly depends on task demand and individual limits, regardless of age. When the memory-load conditions were compared, executive functioning correlated with Updating Working-Memory Task performance at lower load levels in older adults (< 4 updates), and only at higher load levels in young adults (> 3 updates). This result was confirmed by the GLM analysis, showing a significant three-way interaction between age group, load level, and individuals' executive functioning, indicating that executive functioning influenced Updating Working-Memory Task performance differently in young and older adults due to variations in level of task demand. This shows that a higher reliance on executive control in older adults compared to young adults cannot be generalized to all demand levels. Our findings help further our understanding of the assumption that aging is specifically associated with increased reliance on executive control. The shift to controlled forms of processing with advancing age that has been observed in recent studies appears to result from an interacting effect between age and task demand, rather than from aging per se. The significant relationship observed between executive resources and working memory performance under high memory load conditions in young adults confirms that they also call upon executive resources when faced with a particularly demanding task. The main difference with older adults is that younger adults begin to engage executive resources at a higher demand level. Although, in this study, age seems to have a clear effect on the reliance on the executive functioning, given that this reliance also occurs in young adults, our results are better consistent with the idea that this phenomenon is mainly determined by task demand and individual limits than by aging per se.

While the Wisconsin Card Sorting Test-Modified and the Initial Letter-Fluency Test are classically defined as complex tasks capturing some important executive functions, it should be noted that they are also considered as mainly involving a single cognitive flexibility process [33] and thus do not provide a broad assessment of executive abilities. This suggests that we mainly tested reliance on a cognitive flexibility process rather than a more general executive control process. This may limit the scope of this study with regard to the objective of generalizing its conclusion to executive control. This could also apply to the working memory task used in the present study, which captures a single process associated with working memory (i.e., updating). Thus, while the results clearly support the view that reliance on control processes differs in young and older adults as a function of task demand, understanding the relationship between working memory functioning and executive functioning may ultimately require a broader assessment of working memory and executive tasks involving more varied processes.

Secondly, our results help further our understanding of functional neuroimaging data that show patterns of over-activations in older adults relative to young adults, predominantly in brain areas classically associated with executive functioning. They show that the reliance on executive control in young and older adults follows a profile close to the CRUNCH model pattern proposed to account for age-related differences in brain activations (age-related PFC over-activation at low demand level, and under-activation at high demand levels) [17]. Accordingly, our findings are more consistent with this model than with the view that reliance on greater neural resources may be a mechanism specific to the aging brain. Indeed, from a behavioral perspective, our findings seem to parallel functional neuroimaging findings that the involvement of greater frontal neural resources in working memory functioning is mainly dependent on an interacting effect between capacities associated with aging and task demand, rather than on aging per se [19–23]. It is interesting to note that the interaction between age, task demand, and brain activation level observed by some authors also appears for the reliance on executive control. Accordingly, our data may shed further light on why frontal or parietal neural activation increases when faced with greater task demands, suggesting that this phenomenon may correspond to a possible increase in the reliance on executive resources ([16] for a result supporting this view).

The main results of this study are that the reliance on executive control depends on task demand and can be observed in young and older adults at different levels of demand. This finding suggests that the recourse to executive control when performing an Updating Working-Memory Task could be explained better by individual working memory capacity than by aging. Since we observed that the relationship between executive measures and Updating Working-Memory Task performance no longer appears at high demand levels in older adults, one can hypothesize that the engagement of executive resources increases until the limits of memory resources are reached. After a certain threshold of demand in the updating process, it is likely that the difference between older high and low performers on the Updating Working-Memory Task is erased by the difficulty of the task, and hence that individual differences in executive capacities no longer discriminate between these individuals. Older adults are likely to reach their limits at lower levels of demand due to their lower working memory capacity. Regarding neural activation, evidence supporting this hypothesis has been provided by Schneider-Garces et al. [23] using the Sternberg paradigm of working memory. These authors found that the individual level of brain activation associated with task demand is determined by the individual working memory span capacity, irrespective of age. At the cognitive level, further research is required to test the hypothesis that a similar mechanism is involved in the recourse to executive functioning, investigating whether independent measures of short-term or working-memory capacity could be independent main predictors of the relationship between executive control and working-memory performance.

Finally, the greater reliance on controlled processes in older adults has been postulated to be a mechanism to compensate for age-related difficulties [5, 9, 10]. However, two other hypotheses could account for this phenomenon. It could be a simple consequence of the executive function limitation of older adults, placing more effort on these functions without any compensatory purpose, or a result of the aging dedifferentiation phenomenon. The fact that we also found an increase in the reliance on executive measures in young adults at higher load levels does not seem compatible with the dedifferentiation hypothesis, but it does appear consistent with the limitation hypothesis. It should be noted, however, that the limitation and the compensation hypotheses are not mutually exclusive. It would be interesting in a future study to provide arguments in order to explore these two hypotheses.

## References

[1] Cabeza R., & Dennis N. A. Frontal lobes and aging In Stuss DT, Knight RT, editors. Principles of frontal lobes function (pp. 628–652). Oxford University Press, 2012. pp. 628–652.

[2] Diamond A. Executive Functions. Annual Review of Psychology. 2013; 64: 135–168. 10.1146/annurev-psych-113011-143750 23020641 PMC4084861

[3] West R. L. An application of prefrontal cortex function theory to cognitive aging. Psychological Bulletin. 1996; 120: 272–292. 8831298 10.1037/0033-2909.120.2.272

[4] Bouazzaoui B., Fay S., Taconnat L., Angel L., Vanneste S., & Isingrini M. Differential involvement of knowledge representation and executive control in episodic memory performance in young and older adults. Canadian Journal of Experimental Psychology. 2013; 67: 100–107. 10.1037/a0028517 22774803

[5] Bouazzaoui B., Angel L., Fay S., Taconnat L., Froger C. & Isingrini M. Does the greater involvement of executive control in memory with age act as a compensatory mechanism? Canadian Journal of Experimental Psychology, 2014; 68: 59–66. 24364809 10.1037/cep0000005

[6] Glisky E. L., & Kong L. L. Do young and older adults rely on different processes in source memory tasks? A neuropsychological study. Journal of Experimental Psychology: Learning, Memory, and Cognition. 2008; 34: 809–822. 10.1037/0278-7393.34.4.809 18605870 PMC2504728

[7] Hodzik S., & Lemaire P. Inhibition and shifting capacities mediate adults’ age-related differences in strategy selection and repertoire. Acta Psychologica. 2011; 137: 335–344. 10.1016/j.actpsy.2011.04.002 21549334

[8] Jeantin A., & Pennequin V. Explication du déclin du raisonnement inductif par le déficit exécutif lié à l'âge. L'Année Psychologique. 2006; 106: 213–234.

[9] Craik F. I. M., & Rose N. S. Memory encoding and aging: A neurocognitive perspective. Neuroscience and Biobehavioral Reviews. 2012; 7: 1729–1739. 10.1016/j.neubiorev.2011.11.007 22155274

[10] Greenwood P. M., & Parasuraman R. Neuronal and cognitive plasticity: a neurocognitive framework for ameliorating cognitive aging. Frontiers in Aging Neurosciences. 2010; 2: 1–14. 10.3389/fnagi.2010.00150 PMC2999838 21151819

[11] Park D. C., & Reuter-Lorenz P. The Adaptive Brain: Aging and Neurocognitive Scaffolding. Annual Review of Psychology, 2009; 60: 173–196. 10.1146/annurev.psych.59.103006.093656 19035823 PMC3359129

[12] Reuter-Lorenz P. A., Park D. C. How does it STAC up? Revisiting the scaffolding theory of aging and cognition. Neuropsychology Review, 2014; 24: 355–370. 10.1007/s11065-014-9270-9 25143069 PMC4150993

[13] Cabeza R., Daselaar S. M., Dolcos F., Prince S. E., Budde M., & Nyberg L. Task-independent and task-specific age effects on brain activity during working memory, visual attention and episodic retrieval. Cerebral Cortex. 2004; 14: 364–375. 15028641 10.1093/cercor/bhg133

[14] Gutchess A. H., Welsh R. C., Hedden T., Bangert A., Minear M., Liu XX et al Aging and the neural correlates of successful picture encoding: frontal activations compensate for decreased medial-temporal activity. Journal of Cognitive Neurosciences, 2005; 17: 84–96. 10.1162/0898929052880048 15701241

[15] Manenti R., Cotelli M., & Miniussi C. Successful physiological aging and episodic memory: A brain stimulation study. Behavioral Brain Research, 2011; 216: 153–158. 10.1016/j.bbr.2010.07.027 20667492

[16] Angel L., Fay S., Bouazzaoui B. & Isingrini M. Two hemispheres for better memory in old age: role of executive functioning. Journal of Cognitive Neurosciences, 2011; 23: 3676–3777. 10.1162/jocn_a_00104 21812559

[17] Reuter-Lorenz P. A., & Cappell K. A. Neurocognitive aging and the compensation hypothesis. Current Directions in Psychological Sciences, 1008; 17: 177–182.

[18] Bennett I. J., Rivera H. G. & Rypma B. Isolating age-group differences in working memory load-related neural activity: assessing the contribution of working memory capacity using a partial-trial fMRI method. NeuroImage. 2013; 72: 20–32. 10.1016/j.neuroimage.2013.01.030 23357076 PMC3602125

[19] Cappell K. A., Gmeindl L., & Reuter-Lorenz P. A. Age differences in prefrontal recruitment during verbal working memory maintenance depend on memory load. Cortex. 2010; 6: 462–473. 10.1016/j.cortex.2009.11.009 PMC2853232 20097332

[20] Carp J., Gmeindl & Reuter-Lorenz P. A. Age differences in the neural representation of working memory revealed by multi-voxel analysis. Frontiers in Human Neurosciences. 2010; 4: 217. 10.3389/fnhum.2010.00217 PMC2996172 21151373

[21] Mattay V. S., Fera F., Tessitore A., Hariri A. R., Berman K. F., Das S. et al Neurophysiological correlates of age-related changes in working memory capacity. Neuroscience Letters. 2006; 392: 32–37. 16213083 10.1016/j.neulet.2005.09.025

[22] Nagel I. E., Preuschof C., Li SC, Nyberg L., Bäckman L., Lindenberger U et al Load modulation of BOLD response and connectivity predicts working memory performance in younger and older adults. Journal of Cognitive Neuroscience. 2011; 23: 2030–2045. 10.1162/jocn.2010.21560 20828302

[23] Schneider-Garces N. J., Gordon B. A., Brumback-Peltz C. R., Shin E., Lee Y., Sutton B. P., et al, Span, CRUNCH and beyond: Working memory capacity and the aging brain. Journal of Cognitive Neuroscience. 2010; 15: 655–669. 10.1162/jocn.2009.21230 PMC3666347 19320550

[24] Van der Linden M., Brédarts S., & Beerten A. Age-related differences in updating working memory. British Journal of Psychology. 1994; 85: 145–152. 8167975 10.1111/j.2044-8295.1994.tb02514.x

[25] Deltour J.J. Echelle de vocabulaire de Mill Hill de JC Raven Adaptation française et normes comparées du Mill Hill et du Standard Progressive Matrice (PM 38) Manuel. Editions l’application des techniques modernes, Braine-le-Chateau; 1993.

[26] Folstein M. F., Folstein S. E., & McHugh P. R. "Mini-mental state". A practical method for grading the cognitive state of patients for the clinician. Journal of Psychiatric Research. 1975; 12: 189–198. 1202204 10.1016/0022-3956(75)90026-6

[27] Pollack I., Johnson I. B., Knaft P. R. Running memory span. Journal of Experimental Psychology. 1959; 57: 137–146. 13641585 10.1037/h0046137

[28] Morris N., & Jones D. M. Memory updating in working memory: the role of the central executive. British Journal of Psychology. 1990; 81: 111–121.

[29] McCabe D. P., Roediger H. L. III, McDaniel M. A., Balota D. A. & Hambrick D. Z. The relationship between working memory capacity and executive functioning: Evidence for a common executive attention construct. Neuropsychology. 2010; 24: 222–243. 10.1037/a0017619 20230116 PMC2852635

[30] Miyake A., Friedman N. P., Emerson M. J., Witzki A. H., & Howerter A. The unity and diversity of executive functions and their contributions to complex frontal lobe tasks: A latent variable analysis. Cognitive Psychology. 2000; 41: 49–100. 10945922 10.1006/cogp.1999.0734

[31] Souchay C., Isingrini M. & Espagnet L. Aging, episodic feeling-of-knowing and frontal lobe functioning. Neuropsychology. 2000; 14: 1–11. 10.1037//0894-4105.14.2.299 10791869

[32] Nelson H. E. A modified card sorting test sensitive to frontal lobe deficits. Cortex. 1976; 12: 313–324. 1009768 10.1016/s0010-9452(76)80035-4

[33] Lezak M. D. Neuropsychological assessment New York: Oxford University Press; 1995.

[34] Raz N., Gunning-Dixon F. M., Acker J., Head D. & Dupuis J. H. (1998). Neuroanatomical correlates of cognitive aging: evidence from structural magnetic resonance imaging. Neuropsychology. 1998; 12: 95–114. 9460738 10.1037//0894-4105.12.1.95

[35] Arnett P. A., Rao S. M., Bernardins L., Grafman J., Yetkin F. Z. & Lobeck L. Relationship between frontal lobe lesions and Wisconsin Card Sorting Test performance in patients with multiple sclerosis. Neurology. 1994; 44: 420–425. 8145908 10.1212/wnl.44.3_part_1.420

[36] Isingrini M., & Vazou F. Relation between Fluid and frontal lobe functioning in older adults. The International Journal of Aging and Human Development. 1997; 45: 99–109. 9395924 10.2190/WHWX-YNVB-079V-2L74

[37] Stuss D. T., & Benson D. F. The frontal lobes New York: Raven Press; 1986.

[38] Frith C. D., Friston K. J., Liddle P. F., & Frackowiak R. S. Willed action and the prefrontal cortex in man: A study with PET. Proceedings of the National Academy of Sciences. 1991; 244: 241–246. 10.1098/rspb.1991.0077 1679944

[39] Parkin A. J., & Walter B. M. Aging, short-term memory, and frontal dysfunction. Psychobiology. 1991; 19: 175–179.

[40] Troyer A. K, Moscovitch M. & Winocur G. Clustering and switching as two components of verbal fluency: Evidence from younger and older healthy adults. Neuropsychology. 1997; 11: 138–146. 9055277 10.1037//0894-4105.11.1.138

